# Surgical treatment of a giant right atrial myxoma

**DOI:** 10.1186/s40792-020-00923-9

**Published:** 2020-07-08

**Authors:** Ryosuke Numaguchi, Makoto Hashimoto, Ryuji Koshima, Keijiro Mitsube

**Affiliations:** Department of Cardiovascular Surgery, Sapporo Cardiovascular Clinic, North 49, East 16, 8-1, Higashi Ward, Sapporo, Hokkaido 007-0849 Japan

**Keywords:** Cardiac tumor, Right atrial myxoma, Right heart failure

## Abstract

**Background:**

Right atrial myxoma is a rare anomaly. When present, it can rarely cause blood flow obstruction in the right cardiac chamber depending on its size and location. We herein report a rare case of a giant right atrial myxoma, which caused right heart failure due to tricuspid valve obstruction, along with our treatment experience.

**Case presentation:**

A 59-year-old woman was admitted to our hospital for palpitations and edema of the lower extremity. Computed tomography image and transthoracic echocardiography showed a 57 mm × 63 mm giant tumor obstructing the tricuspid valve inflow. The tumor was excised surgically and the residual septal defect was repaired with a Dacron patch. Tricuspid valve annuloplasty was performed additionally. The postoperative course was uneventful and the patient was discharged on postoperative day 5.

**Conclusion:**

Giant right atrial myxoma is a rare cause of tricuspid valve stenosis and right heart failure. Surgical resection is the most appropriate treatment option and should be performed as soon as possible after diagnosis.

## Background

Cardiac myxoma is the most common type of primary heart tumor. Although most myxomas are pathologically benign tumors, they can cause embolic symptoms, obstructive symptoms, and even sudden cardiac death depending on the location and size of the mass. Therefore, symptomatic myxomas should be excised surgically as soon as possible [1]. Over 75% of myxomas are found in the left atrium. Conversely, right atrial myxoma is relatively rare and accounts for less than 15% of all myxomas [1]. Furthermore, a symptomatic giant right atrial myxoma is rarely reported. Herein, we present a rare case of symptomatic giant right atrial myxoma which caused right heart failure by obstructing intra-cardiac circulation.

## Case presentation

**Fig. 1:**
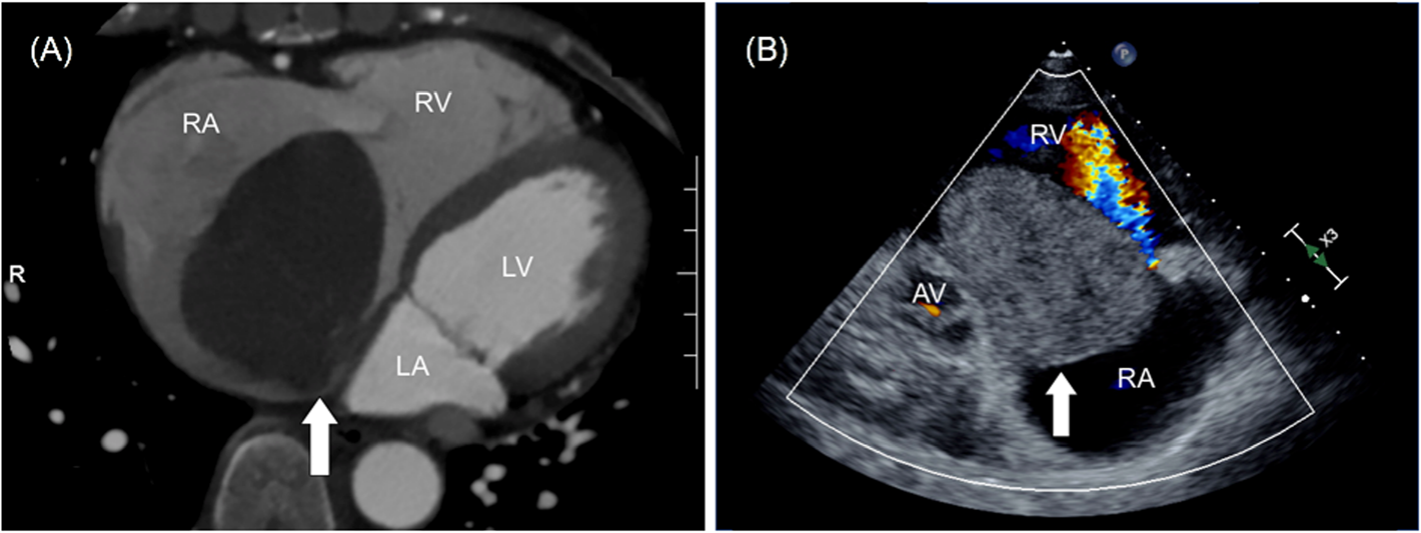
**a** CT showing a large mass in the right atrium (white arrow). **b** Preoperative TTE showing a 57 mm × 63 mm giant tumor obstructing the tricuspid valve inflow (white arrow). RA, right atrium; RV, right ventricle; LA, left atrium; LV, left ventricle; AV, aortic valve

A 59-year-old woman (height, 151 cm; weight, 57 kg; body surface area, 1.52 m^2^) was referred to our hospital for palpitations and edema of the lower extremity. The patient had no significant medical history. Electrocardiography revealed a heart rate of 76 beats/min and first-degree atrioventricular block. Liver function was almost normal with an aspartate aminotransferase (AST) level of 40 U/L and alanine aminotransferase (ALT) level of 25 U/L. Renal function was normal with a creatinine level of 0.56 mg/dL. Computed tomography (CT) image showed a pedunculated mobile tumor arising from the interatrial septum (Fig. 1a). Blood flow obstruction inside the right heart was suspected by a 4-dimensional CT (Video 1). Transthoracic echocardiography (TTE) showed a 57 mm × 63 mm giant tumor obstructing the tricuspid valve inflow in each cardiac cycle (Fig. 1b, Video 2). As a result, the tumor mimicked tricuspid stenosis with a mean trans-tricuspid pressure gradient of 5 mmHg. The diagnosis of right atrial myxoma associated with congestive right heart failure was confirmed, and urgent surgical resection was scheduled 1 day after diagnosis.

**Additional file 1: Video 1.** Preoperative 4D CT showing giant right atrial myxoma prolapsing through the tricuspid annulus.

**Additional file 2: Video 2.** Preoperative TTE showing a giant tumor obstructing the tricuspid valve inflow.

After a median sternotomy and aortic cross-clamping, the right atrium was opened through an oblique atriotomy. The huge mobile tumor was attached to the edge of the fossa ovalis and was resected together with the atrial septum to which the tumor stalk was attached. The residual septal defect was repaired with a Dacron patch (Sauvage Filamentous Knitted Polyester Fabric, Bard Peripheral Vascular Inc., AZ, USA). The tricuspid valve leaflets and subvalvular apparatus seemed normal and were left without any surgical intervention. However, transesophageal echocardiography (TEE) showed moderate tricuspid valve regurgitation due to annular dilatation while weaning from cardiopulmonary bypass (CPB). Aortic cross-clamping was repeated, and tricuspid valve annuloplasty was performed using a 30-mm tricuspid annuloplasty ring (Physio Tricuspid annuloplasty ring, Edwards Lifesciences Co., Tokyo, Japan). The size of the ring was based on the length of the septal annulus. The duration of CPB was 68 min and the total cardiac arrest time was 32 (20 + 12) min.

**Fig. 2:**
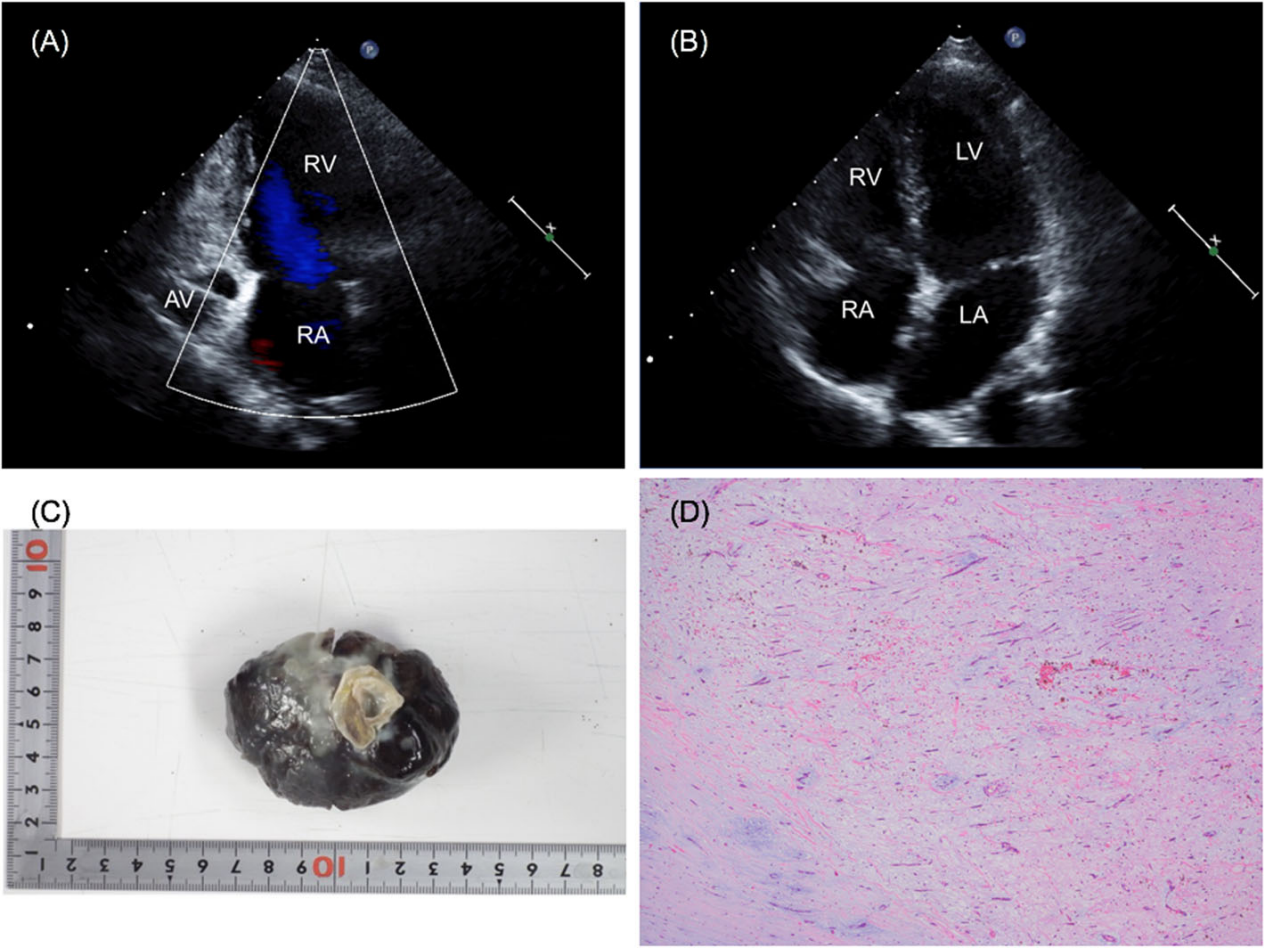
**a**, **b** Postoperative TTE showing complete removal of the tumor. AV, aortic valve; RA, right atrium; RV, right ventricle; LA, left atrium; LV, left ventricle. **c** Excised elastic hard tumor measuring 7.0 × 5.0 × 3.0 cm. **d** Histologic image showing myxoid stroma (hematoxylin and eosin stain)

Postoperative TTE showed no abnormal findings with well-controlled tricuspid regurgitation (Fig. 2a, b). The histopathological examination revealed benign myxoma with myxoid stroma and hemorrhagic necrosis (Fig. 2c, d). The postoperative course was uneventful and the patient was discharged on postoperative day 5.

## Discussion

Cardiac myxoma is the most common benign primary heart tumor. Although it can occur within any cardiac chamber, a majority of them are located within the left atrium. Right atrial myxoma is a relatively rare anomaly as it only accounts for about 15–20% of all myxoma cases. Typically, atrial myxomas, whether left or right, arise from the interatrial septum, usually at the border of the fossa ovalis [1]. Depending on the size, location, and mobility of the mass, myxomas give rise to 3 types of symptoms: (1) hemodynamic derangement due to flow obstruction within the cardiac chambers, (2) symptoms due to tumor embolization, and (3) constitutional symptoms [2]. Furthermore, if the tumor is large and mobile enough, complete obstruction of the orifice of the valve can occur, resulting in sudden death [3]. Therefore, symptomatic myxomas should be excised surgically as soon as possible after diagnosis.

The surgical prognosis of cardiac myxoma is excellent with a mortality rate of less than 3% [1]. According to Jones and colleagues, the surgical approach for atrial myxomas should (1) allow minimal manipulation of the tumor, (2) provide adequate exposure for complete resection of the mass, (3) allow inspection of all four heart chambers, and (4) be safe and efficacious [4]. They reported excellent surgical results by employing a bi-atrial approach for all cases, mostly by a median sternotomy.

Conversely, a minimum invasive approach for extirpation of cardiac tumor has been recently reported with favorable outcomes [5]. In cases of low-risk, small mass, single surgical procedure, the minimum invasive approach should be considered as a feasible treatment option. Furthermore, recent development of diagnostic imaging provides accurate preoperative evaluation of the tumor size and location. Therefore, inspection of all four heart chambers may not be required in all cases.

In this case, we performed an additional tricuspid annuloplasty. Tricuspid valve regurgitation was not detected preoperatively; however, the tumor size was large enough to dilate the tricuspid annulus. Therefore, tricuspid annuloplasty should have been considered preoperatively.

## Conclusion

Giant right atrial myxoma is a rare cause of tricuspid valve stenosis and right heart failure. Surgical resection is the most appropriate treatment option and should be performed as soon as possible after diagnosis.

## Data Availability

Data sharing is not applicable to this article as no datasets were generated or analyzed during the current study.

## Abbreviations

CT: Computed tomography
TTE: Transthoracic echocardiography
AST: Aspartate aminotransferase
ALT: Alanine aminotransferase
CPB: Cardiopulmonary bypass
TEE: Transesophageal echocardiography
